# Untargeted Metabolomics Uncovers the Essential Lysine Transporter in *Toxoplasma gondii*

**DOI:** 10.3390/metabo11080476

**Published:** 2021-07-23

**Authors:** Joachim Kloehn, Matteo Lunghi, Emmanuel Varesio, David Dubois, Dominique Soldati-Favre

**Affiliations:** 1 Department of Microbiology and Molecular Medicine, University of Geneva, CMU, Rue Michel-Servet 1, 1211 Geneva, Switzerland; matteo.lunghi@unige.ch (M.L.); daviddubois27@protonmail.com (D.D.); 2 Institute of Pharmaceutical Sciences of Western Switzerland, School of Pharmaceutical Sciences, Mass Spectrometry Core Facility (MZ 2.0), University of Geneva, 1211 Geneva, Switzerland; Emmanuel.Varesio@unige.ch

**Keywords:** *Toxoplasma gondii*, Apicomplexa, obligate intracellular parasite, amino acids, lysine, transporter, solute carrier, major facilitator superfamily, metabolomics, untargeted metabolomics, stable isotope labeling

## Abstract

Apicomplexan parasites are responsible for devastating diseases, including malaria, toxoplasmosis, and cryptosporidiosis. Current treatments are limited by emerging resistance to, as well as the high cost and toxicity of existing drugs. As obligate intracellular parasites, apicomplexans rely on the uptake of many essential metabolites from their host. *Toxoplasma gondii*, the causative agent of toxoplasmosis, is auxotrophic for several metabolites, including sugars (e.g., myo-inositol), amino acids (e.g., tyrosine), lipidic compounds and lipid precursors (cholesterol, choline), vitamins, cofactors (thiamine) and others. To date, only few apicomplexan metabolite transporters have been characterized and assigned a substrate. Here, we set out to investigate whether untargeted metabolomics can be used to identify the substrate of an uncharacterized transporter. Based on existing genome- and proteome-wide datasets, we have identified an essential plasma membrane transporter of the major facilitator superfamily in *T. gondii*—previously termed TgApiAT6-1. Using an inducible system based on RNA degradation, TgApiAT6-1 was depleted, and the mutant parasite’s metabolome was compared to that of non-depleted parasites. The most significantly reduced metabolite in parasites depleted in TgApiAT6-1 was identified as the amino acid lysine, for which *T. gondii* is predicted to be auxotrophic. Using stable isotope-labeled amino acids, we confirmed that TgApiAT6-1 is required for efficient lysine uptake. Our findings highlight untargeted metabolomics as a powerful tool to identify the substrate of orphan transporters.

## 1. Introduction

**Figure 1:**
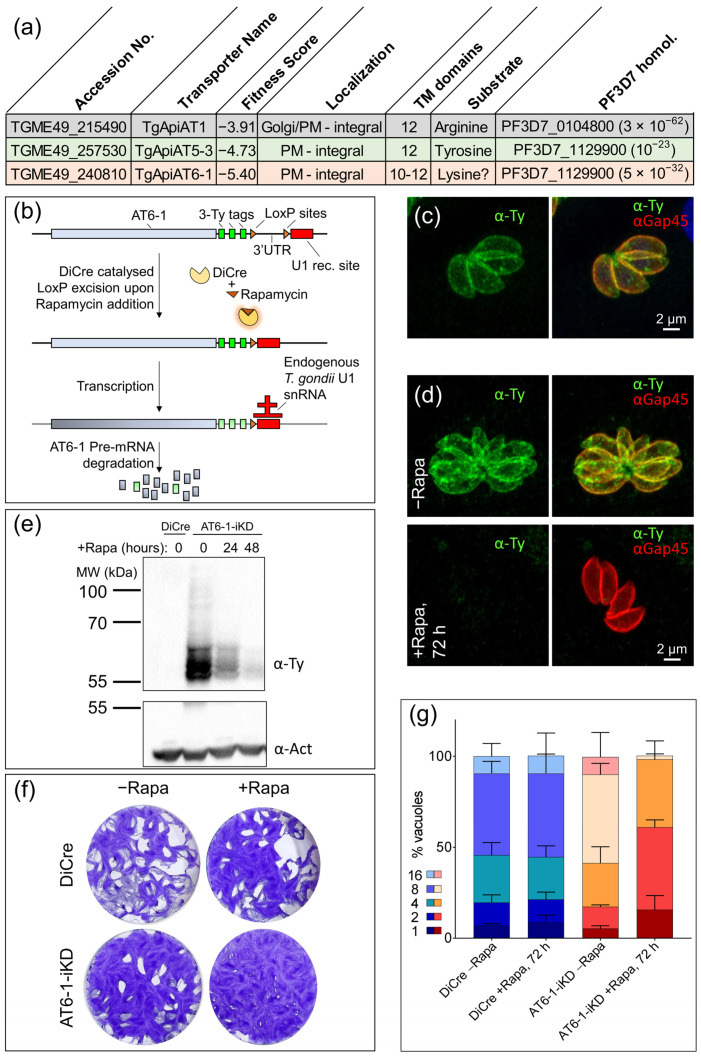
AT6-1 is an essential plasma membrane transporter. (**a**) Overview of known amino acid transporters in *T. gondii*. TgApiAT1 and TgApiAT5-3 were identified as arginine and tyrosine transporters, respectively [11,12]. TgApiAT6-1 was recently putatively characterized as a lysine transporter [26,27]. (**b**) Cartoon scheme of AT6-1 locus modification by introducing a 3-Ty-tag, loxP sites and a U1 recognition site, enabling localization of AT6-1 and its conditional down-regulation. (**c**) Immunofluorescence assay (IFA) to determine the localization of AT6-1 (Ty, green) with the pellicle marker GAP45 as a reference for the cell periphery (red). (**d**) IFA of AT6-1 control and following 72 h of rapamycin treatment. (**e**) Western blot showing signal for AT6-1 (Ty) after 24 and 48 h of rapamycin treatment. Antibodies against actin were used as a loading control (α-Act). (**f**) Lysis plaques of parasites lacking AT6-1 (+Rapa) and controls following one week of growth. (**g**) Intracellular growth assay of parasites depleted in AT6-1 and controls. Growth (number of parasites per vacuole) was assessed over 24 h, following 48 h of rapamycin pre-treatment (72 h rapamycin total).

The apicomplexans form a large phylum comprising thousands of obligate intracellular parasites, some of which infect humans and cause devastating diseases, including malaria, cryptosporidiosis and toxoplasmosis. *Toxoplasma gondii*, the causative agent of toxoplasmosis, infects about one third of the human population [1,2]. Although infection is typically asymptomatic, the uncontrolled replication of the fast-replicating tachyzoite stage in immunocompromised individuals, including HIV patients and organ transplant recipients, can have severe consequences, including encephalitis and necrotizing retinochoroiditis [3]. Furthermore, the parasite poses a risk to the fetus if primary infection occurs during pregnancy, as it can pass the placenta, causing severe health problems for the newborn or even leading to abortion or still birth [4]. In healthy individuals, upon immune pressure, the parasite differentiates to slow-growing so-called bradyzoites, which evade the immune system and persist encysted in smooth muscle cells and neurons for the lifetime of their host [5]. As obligate intracellular parasites, apicomplexans are experts at salvaging essential metabolites from their host [6]. The efficient uptake of metabolites depends on transporters embedded within the plasma membrane [7,8]. *T. gondii* is auxotrophic for several metabolites that must be acquired through transporters, most of which have not been characterized to date [6]. The few characterized transporters for the import of vital metabolites include the parasite’s glucose transporters [9], a transporter for adenosine [10], the essential amino acids tyrosine and arginine [11,12], as well as folate [13]. The recently identified transporters identified for tyrosine and arginine belong to the major facilitator superfamily (MFS) (Figure 1a), which are characterized by two structurally similar domains that form 12 transmembrane domains, carry typically small solutes and possess a common motif between the second and third transmembrane spanner [14,15]. *T. gondii* encodes approximately 18 to >30 MFS transporters [7,16], several of which are predicted to be essential based on a genome-wide CRISPR/Cas9 fitness screen [17]. MFS transporters are found within the plasma membrane as well as within the membranes of parasite organelles, including the essential transporter facilitator protein 1 (TFP1), which localizes to the micronemes and is required for the maturation of this secretory organelle critical for motility, invasion and egress from host cells [16]. Aside from the tyrosine and arginine transporters, the substrates of other MFS transporters are not known. Here, we identified an essential plasma membrane transporter and employed untargeted metabolomics to uncover the Apicomplexan Amino Acid Transporter 6-1 (TgApiAT6-1, in the following referred to as AT6-1) [11] as the essential lysine transporter in *T. gondii* tachyzoites.

## 2. Results

Amongst *T. gondii* MFS transporters, one of the lowest fitness scores (−5.4) was assigned to TGME49_240810 in a genome-wide CRISPR/Cas9 screen, indicating its importance for the parasite’s lytic cycle [17]. A recent study, which utilized the hyperplexed localization of organelle proteins by isotope tagging (hyperLOPIT) to assign the subcellular localization to approximately 3000 *T. gondii* proteins, localized TGME49_240810 to the parasite plasma membrane [18]. Two additional studies have previously localized endogenously tagged TGME49_240810 to the plasma membrane [11,19]. While *T. gondii* secretes some metabolic end products across the plasma membrane, including lactate, alanine, and succinate, the list of secreted metabolites is relatively short, and the lactate transporters have been identified and characterized [20,21,22]. Thus, TGME49_240810, here referred to as AT6-1 [11], was considered a strong candidate for a transporter facilitating the uptake of an essential metabolite across the parasite plasma membrane. To localize AT6-1 and allow its conditional down-regulation, an epitope tag (Ty) was fused to its endogenous locus (Figure 1b) and loxP sites were simultaneously inserted after the stop codon of the endogenous locus, followed by a U1 recognition site in the 3′-untranslated region (3′-UTR) (Figure 1b). This modification at the AT6-1 locus was mediated by CRISPR/Cas9 editing in a parasite line that stably expresses dimerizable Cre recombinase (DiCre) [23], as previously described [24,25] (Figure 1b), using primers listed in Table S1. Clones from the transfected population were picked and successful integration of the construct was validated by PCR, amplifying a segment of genomic DNA with primers that anneal within the AT6-1 gene locus and within the hypoxanthine–xanthine–guanine phosphoribosyl transferase (HXGPRT) resistance cassette (Figure 1a, Supplementary Table S1). In an immunofluorescence assay (IFA), the Ty-tag fused to AT6-1 partially colocalized with gliding-associated protein 45 (GAP45), a pellicle marker, consistent with the localization of AT6-1 at the cell periphery, most likely the plasma membrane (Figure 1c).

Due to the inserted loxP sites, the addition of rapamycin (50 nM) results in excision of the 3′-UTR, causing the U1 recognition site to be placed directly after the stop codon. The transcribed RNA is degraded, resulting in a conditional knock-down of the gene of interest (AT6-1-iKD). The conditional down-regulation of AT6-1 was confirmed by IFA (Figure 1d) and Western blot (Figure 1e) following addition of 50 nM rapamycin for different time periods as indicated.

To investigate the importance of AT6-1 in the lytic cycle of *T. gondii*, we performed plaque assays culturing the AT6-1-iKD strain and its parental line in the presence of rapamycin (50 nM, +R) or its absence (−R) for one week. The monolayer of human foreskin fibroblasts (HFFs) was stained with crystal violet, revealing plaques of lysis. A severe reduction in plaque size was observed for AT6-1-iKD+R parasites, confirming the transporter’s crucial role for the parasite’s lytic cycle (Figure 1f). To test if this defect in the lytic cycle is due to a growth defect, we performed an intracellular growth assay, counting the number of parasites per vacuole after 24 h of growth and following 72 h of rapamycin treatment. A severe defect in intracellular growth was observed in AT6-1-iKD+R (Figure 1g), further confirming the importance of AT6-1 for parasite fitness and replication.

**Figure 2:**
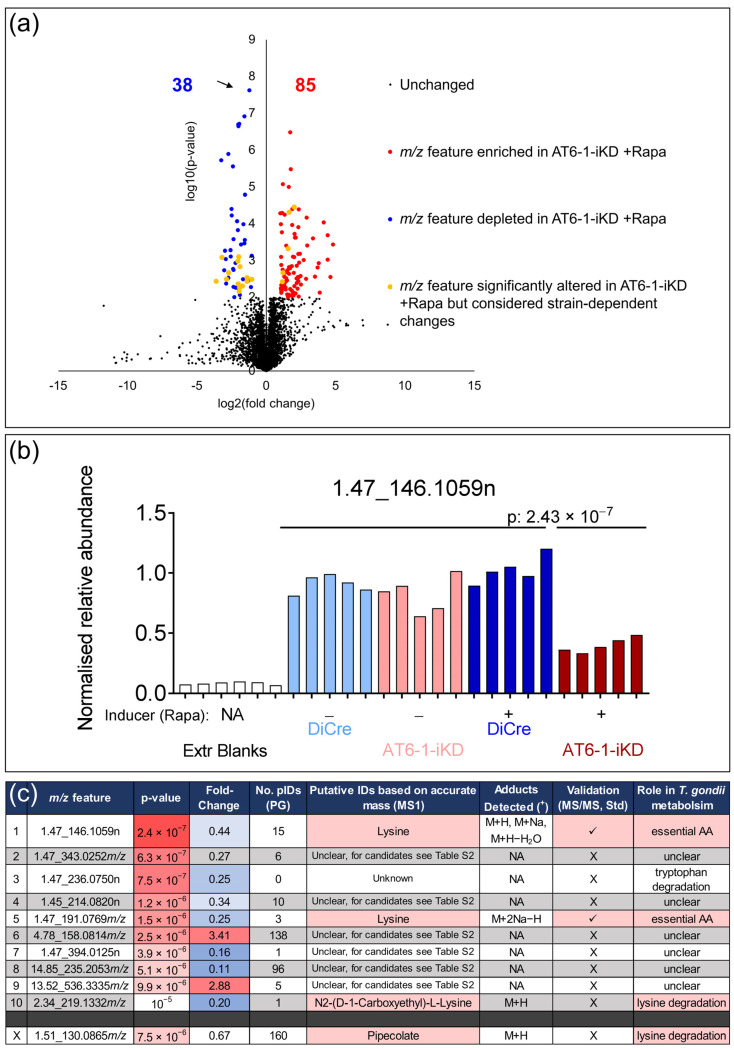
Untargeted metabolomic analysis of *T. gondii* depleted in AT6-1. (**a**) Volcano plot highlighting the mass features that changed significantly in their abundance (*p* < 0.01, fold change > 2) down-regulation of AT6-1 through rapamycin (+R) (2-sided Student’s t-test comparing AT6-1-iKD+R vs. AT6-1-iKD−R/DiCre+/−R). Inducer-dependent changes (23, yellow) and strain-dependent changes (0) were filtered out, as outlined in the text. (**b**) The most significantly altered metabolite (*p* = 2.43 × 10^−7^) in AT6-1-iKD+R parasites (see arrow in (**a**)) was a mass feature with a neutral mass of 146.1059 eluting at 1.47 min. Its normalized abundance is shown for each independent biological replicate and the extraction blanks. (**c**) A list of the 10 most significantly altered mass features in AT6-1-iKD+R is shown. The *p*-value and fold-change when comparing AT6-1-iKD+R vs. AT6-1-iKD−R/DiCre+R/DiCre−R are given. The number of putative identifications in Progenesis QI v.2.4 (Waters-NonlinearDynamics) are shown. The most significantly altered feature and the 5th most significantly altered feature were attributed to the essential amino acid lysine. Two additional mass features were putatively attributed to the lysine degradation products (N2-(D-1-carboxyethyl)-l-lysine and pipecolate.

Next, untargeted metabolomics were employed to characterize the function of AT6-1. To identify the substrate of AT6-1 and to avoid major downstream effects on the metabolome, such as a general stress response due to low levels of the metabolite of interest, we decided to screen the parasite’s metabolome after 24 h of down-regulation. By Western blot, we had observed the considerable, but incomplete, down-regulation of AT6-1 at that time point (Figure 1e). Following 24 h of rapamycin treatment (50 nM), five replicates of freshly egressing parasites (2 × 10^8^ parasites, parental line DiCre+/−R, AT6-1-iKD+/−R) were harvested and metabolites extracted. Samples were analyzed by reverse-phase ultrahigh performance liquid chromatography mass spectrometry tandem mass spectrometry (UHPLC-MS/MS) in positive mode. Mass features significantly altered in abundance upon the loss of AT6-1 were determined by comparing the normalized relative abundances of each mass feature in parasites depleted in AT6-1 (AT6-1-iKD+R) to that in all other control samples (DiCre+/−R, AT6-1-iKD−R) using a two-sided Student’s *t*-test. Metabolites of interest were defined as those that were altered statistically significantly (*p* < 0.01) and displayed >2-fold change (FC) in abundance. Table S2 shows all ~4800 mass features sorted by their *p*-value, comparing AT6-1-iKD+R vs. controls. A total of 85 mass features were found to be significantly increased in AT6-1-iKD+R (*p* < 0.01, FC > 2), while 38 were significantly decreased (*p* < 0.01, FC > 2) (Figure 2a, Table S2). Amongst the metabolites significantly altered in AT6-1-iKD+R, we considered whether these were specifically due to AT6-1 down-regulation or if they could instead be considered strain differences (more significantly changed in DiCre+/−R vs. AT6-1-iKD+/−R) or due to other effects of the inducer rapamycin (more significantly changed in DiCre+R/AT6-1-iKD+R vs. DiCre−R/AT6-1-iKD−R). Of the metabolites significantly altered in AT6-1-iKD+R, 23 mass features were found more significantly altered between the strains, and none for the inducer rapamycin (Figure 2a, Table S2).

To identify the substrate of AT6-1, the 10 most significantly altered metabolites in AT6-1-iKD+R were considered. The ‘top-hit’ with a neutral mass of 146.1059 eluting at 1.47 min was reduced to a relative average abundance of 0.43, specifically in AT6-1-iKD+R compared to a normalized abundance of 1 in the controls (Figure 2b). This mass feature matched 15 putative identifications using the library search feature in Progenesis QI v.2.4 (Waters-NonlinearDynamics) software based on accurate mass (Table S2, Figure 2c). Amongst the candidate metabolites was lysine, an essential amino acid for *T. gondii* [28]. The MS/MS spectrum and analysis of an authentic standard further confirmed this metabolite as lysine. Another mass feature amongst the top 10 was identified as the uncommon M+2Na−H adduct of lysine, which was initially not included in the list of expected adducts in Progenesis QI (Figure 2c). While most other mass features had several putative IDs, which could not be linked to known metabolites in *T. gondii*, two putatively identified compounds were linked to lysine metabolism: the putative lysine degradation product N2-(d-1-carboxyethyl)-l-lysine and pipecolate (Figure 2c, Table S2). The latter compound was described in several metabolomic analyses of apicomplexan parasites [29,30,31]. Lysine and its related metabolites were all decreased in abundance, consistent with AT6-1 potentially functioning as a lysine transporter. Pipecolate, however, was not in the top 10 list as it was significantly reduced in abundance but not >2-fold (0.67 relative abundance in AT6-1-iKD+R, compared to 1 in controls). Although not further investigated here, the other changes observed in AT6-1-iKD+R could potentially be secondary effects following the lack of lysine, such as increased stress metabolites associated with impeded protein synthesis or a decrease in lysine-containing short peptides.

**Figure 3:**
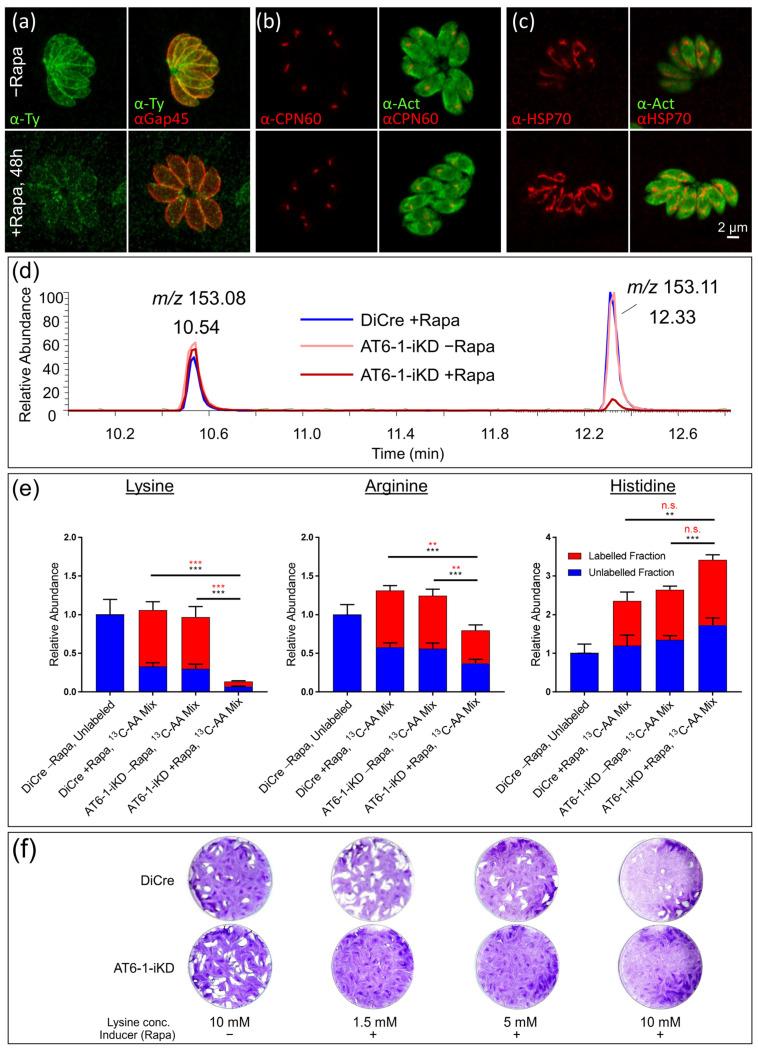
Validation of AT6-1 as the *T. gondii* lysine transporter. (**a**–**c**) Immunofluorescence assays (IFAs) of AT6-1-iKD without (top panels) or with rapamycin for 48 h (bottom panels). For antibodies, see top panels and for scale bar, see (**c**). IFA of Ty-tagged AT6-1 (green) and pellicle marker GAP45 (red) (**a**). IFA of apicoplasts (chaperone 60, CPN 60, red) and actin (Act, green) (**b**). IFA of mitochondrion (heat shock protein 70, HSP70, red) and actin (Act, green) (**c**). (**d**) Representative ion chromatogram of parasite extracts. Parasites were treated with rapamycin (+R) or its absence (−R) for 48 h and incubated in medium containing a mix of U-^13^C-labeled amino acids. The panel shows a representative chromatogram (mass spectrum *m/z* 153.05–153.15) showing the signal for U-^13^C-glutamate (*m/z* 153.08, eluting at 10.54 min) and U-^13^C-lysine (*m/z* 153.13, eluting at 12.33 min) in AT6-1-iKD-/+R and in DiCre+R parasites. (**e**) The relative abundance and fractional ^13^C-labeling of the 3 positively charged amino acids (lysine, arginine and histidine) are shown for DiCre-/+R parasites and AT6-1-iKD-/+R parasites. DiCre−R parasites were incubated in regular (natural abundance) medium, while AT6-1-iKD-/+R parasites and DiCre+R parasites were incubated in medium containing a mix of U-^13^C-labeled amino acids for 1 h. The abundance was determined as the sum of each amino acid’s mass isotopologues normalized to the internal standard and to the relative abundance in DiCre−R parasites (normalized abundance of 1). Based on the abundance of all mass isotopologues following background correction, ^13^C-labeling was quantified. Statistically significant differences in abundance (bottom, black) and ^13^C-labeling (top, red) are indicated (not significant: n.s., **: *p*-value < 0.001, ***: *p*-value < 0.0001). (**f**) Plaque assays of AT6-1-iKD parasites −/+R in the presence of different lysine concentrations (1.5 mM, 5 mM and 10 mM).

To investigate whether AT6-1 may indeed function as a lysine transporter, an amino acid uptake assay was performed using a U-^13^C amino acid mix. Parasites were harvested as described above, following the down-regulation of AT6-1 through 50 nM rapamycin for 48 h. Purified and washed parasite pellets (five replicates per condition) were resuspended in regular medium (DiCre−R) or in medium supplemented with a U-^13^C amino acid mix (DiCre+R, AT6-1-iKD−R/+R) and incubated for 1 h. Parasites were assessed by IFA to confirm the efficient down-regulation of AT6-1 over 48 h of rapamycin treatment. The signal for Ty was markedly decreased, but parasites appeared morphologically normal and intact (Figure 3a). Similarly, we evaluated the morphology of the mitochondrion and the apicoplast, two crucial metabolic compartments in *T. gondii*. Both organelles appeared intact and morphologically normal (Figure 3b,c), indicating that parasites are viable at the time point of analysis.

Rather than using reverse-phase separation (RPLC) as above, metabolites were analyzed by UHPLC-MS/MS using hydrophilic interaction chromatography (HILIC), better suited for the separation of polar compounds such as amino acids. Amino acids were identified based on their accurate mass and retention time of authentic standards (U-^13^C amino acid mix). A dramatic reduction was observed in the uptake of the essential amino acid lysine, as indicated by low levels of U-^13^C_6_-lysine (Figure 3d,e). The abundance of some essential amino acids (histidine, phenylalanine, tyrosine) was slightly increased in the labeling experiment, likely owed to the higher abundance of amino acids in the medium (Figure 3e, Figure S1b). Strikingly, the abundance of lysine was 7.7-fold reduced in AT6-1+R parasites compared to the controls. Additionally, the percentage of labeling was significantly reduced, even in this decimated lysine pool, from 69% to 48%. These findings are consistent with AT6-1 functioning as a lysine transporter (Figure 3e).

The abundance and labeling of arginine were also modestly (1.5-fold) but significantly reduced in AT6-1-iKD+R parasites compared to AT6-1-iKD−R and DiCre+R parasites, indicating that AT6-1 might play a minor role as an arginine transport, as recently postulated [27] (Figure 3e). In contrast, histidine levels were slightly but significantly elevated in AT6-1-iKD+R parasites compared to AT6-1-iKD−R and DiCre+R parasites, while the labeling fraction was unaffected. Other amino acids were not significantly altered in abundance or in labeling upon the down-regulation of AT6-1 (Figure S1b). The relative abundance and fraction of labeling are representatively shown for a negatively charged amino acid (aspartate), a polar amino acid (tyrosine) and a nonpolar amino acid (phenylalanine) (Figure S1b). Tyrosine and phenylalanine are essential for *T. gondii* [11,32], while aspartate can be synthesized in the TCA cycle [22]. This endogenous production is reflected in the low level of labeling from exogeneous aspartate (Figure S1b).

Finally, to test if the loss of AT6-1 could be rescued by high levels of exogenous lysine, through uptake via a low affinity transporter, plaque assays were carried out as described above, but culturing parasites in the presence of 1.5, 5 or 10 mM of lysine in the media (Figure 3f). Even very high levels of exogeneous lysine did not rescue the defect of AT6-1-iKD+R, indicating that AT6-1 is the sole lysine transporter in *T. gondii* tachyzoites.

## 3. Discussion

Obligate intracellular parasites must salvage numerous metabolites from their host to survive and replicate [6]. The controlled uptake of metabolites depends on transmembrane transporter proteins. Despite their critical role in the parasite’s metabolism and homeostasis, only a fraction of transporters have been identified and characterized. The critical role of metabolite transporters and their potential as drug targets in parasitic protozoa are increasingly recognized [7,8,33,34]. The requirements for amino acids and uptake of other metabolites likely differ between tachyzoites and the encysted bradyzoites, but these differences are poorly defined to date [35]. Similar to its host, the apicomplexan parasite *T. gondii* relies on the uptake of several essential amino acids [28]. While *T. gondii* surprisingly encodes few enzymes which may function in the synthesis pathway of lysine, the pathway is considered incomplete, making the parasite auxotrophic for this basic amino acid [28]. Previously, Parker et al. and Rajendran et al. have identified several putative amino acid transporters belonging to the MFS that were termed Apicomplexan Amino Acid Transporters (ApiATs) [11,12]. ApiAT1, previously named novel putative transporter 1 (NPT1), was shown to transport arginine [12], while ApiAT5-3 was identified as the transporter of tyrosine [11]. The growth defect in parasites lacking NPT1 was severe in medium containing low levels of arginine but could be fully compensated for through high levels of exogenous arginine [12]. These findings suggested that NPT1 functions as an arginine transporter but that an additional arginine transporter exists, which facilitates the transport of arginine with a lower affinity. Recently, AT6-1 (TGME49_240810) was proposed as the lysine transporter and low affinity arginine transporter [26,27]. These amino acid transporters were identified based on targeted or semi-targeted analyses using stable isotope labeled amino acids. To characterize the role of AT6-1, we instead utilized untargeted metabolomics to identify which metabolite(s) change(s) significantly upon the down-regulation of the transporter. The most significantly altered (decreased) metabolite in AT6-1-depleted parasites was identified as lysine. In contrast to NPT1, a lack of AT6-1 could not be rescued by providing high levels of exogenous lysine, indicating that AT6-1 is the sole lysine transporter of *T. gondii*. We further observed a modest but significant decrease in arginine uptake upon the down-regulation of AT6-1, consistent with its role as a low affinity arginine transporter [26,27]. To the best of our knowledge, this is the first study employing untargeted metabolomics to characterize an apicomplexan solute carrier. This unbiased approach is suitable for large-scale screens and paves the way for future studies to decipher the ‘transportome’ of apicomplexans.

## 4. Materials and Methods

### 4.1. Parasite Culture and Maintenance

The *T. gondii* DiCre strain was a generous gift from Moritz Treeck. Parasites were maintained under standard tachyzoite growth conditions through regular passage on a confluent monolayer of human foreskin fibroblasts (HFFs) in Dulbecco Modified Eagle Medium (DMEM, Gibco, 41966-029) supplemented with 5% fetal bovine serum (FBS, Gibco, 10270-106), 2 mM L-glutamine (Gibco, 20530-024) and 25 μg/mL gentamycin (Gibco, 15750-045). Cultures were maintained in humidified incubators at 37 °C and 5% CO_2_.

### 4.2. Cloning of DNA Constructs

The AT6-1-iKD strain was generated by co-transfection of a CRISPR-Cas9 expression plasmid [36] (primer P1) targeting the 3′-UTR of the gene, and a PCR-generated (primers P2 and P3) homology repair template encoding the 3-Ty U1 tagging construct [23]. Primers are listed in Table S1. Transfected parasites were selected for 1 week with 25 μg/mL mycophenolic acid and 50 μg/mL xanthine for HXGPRT positive selection [37], and stable transfectants were cloned by serial dilution.

### 4.3. Genomic DNA Extraction and Integration PCR

Genomic DNA was extracted from the DiCre parental line and AT6-1-iKD parasites using the Wizard Genomic DNA Purification Kit (Promega, Madison, WI, USA). Integration of the construct in the AT6-1 locus was confirmed by integration PCR using the primers P4 and P5 (see Table S1) and the GoTaq DNA Polymerase (Promega, Madison, WI, USA), generating a fragment of 1267 bp. The PCR program was as follows: 95 °C, 2 min; (95 °C, 15 s; 57 °C 15 s; 72 °C 1.5 min) × 35; 72 °C, 5 min on an Applied Biosystems (Waltham, MA, USA), SimpliAmp Thermal Cycler.

### 4.4. Plaque Assays and Intracellular Growth Assays

Plaque assays and growth assays were carried out as described previously [21,38]. In brief: for plaque assays, different dilutions of parasite cultures were incubated on a confluent host cells monolayer for 7 days. After 7 days, the infected monolayer was gently washed with PBS and fixed with 4% paraformaldehyde (PFA) for 10 min. The monolayer was again washed with PBS prior to staining with a crystal violet solution (12.5 g crystal violet, 125 mL ethanol mixed with 500 mL water containing 1% (*w*/*v*) ammonium oxalate). Plaque assays were washed 3 times with deionized water to remove excess crystal violet and visualize plaques.

For growth assays, confluent HFFs grown on coverslips were inoculated with 10 μL of a freshly lysing culture. Parasites were pre-treated with rapamycin and treated with rapamycin during the growth assay as indicated. Twenty-four hours after inoculation, parasites were fixed with 4% PFA and 0.05% glutaraldehyde for 10 min, prior to quenching with 0.1 M glycine in PBS. Infected host cells were permeabilized (0.2% Triton X-100/PBS), blocked (2% BSA/0.2% Triton X-100/PBS) and probed with an antibody against the pellicle marker GAP45 diluted in 2% BSA/0.2% Triton X-100/PBS for 1 h (polyclonal rabbit anti GAP45 (1:10,000) [39]). The probed monolayer was washed (3 × 5 min, 0.2% Triton X-100/PBS) and probed with a secondary antibody prior to washing as for the primary antibody (anti-rabbit Alexa fluor 594 (Invitrogen A11012). The coverslip was mounted on microscopy slides using FluoromountG. Slides were viewed on a Nikon Eclipse Ti inverted microscope (Nikon, Minato, Japan). The number of parasites was counted in >100 vacuoles per condition and replicated for 3 independent biological replicates.

### 4.5. Immunofluorescence Assays

IFAs were performed using antibodies and acquiring and processing images as described previously [21,38] and as described above for the growth assay. Antibodies used in the study (used dilution): polyclonal rabbit anti GAP45 (1:10,000) [39], monoclonal mouse α-actin (1:20) [40], α-Ty (1:10, BB2). Secondary anti mouse Alexa fluor 488 (Invitrogen, Waltham, MA, USA; A11001), anti-rabbit Alexa fluor 594 (Invitrogen, Waltham, MA, USA; A11012), polyclonal rabbit α-chaperone 60 (CPN60, 1:3000) [41], polyclonal rabbit α-heat shock protein 70 (HSP70, 1:3000) [42]. Images were acquired using an LSM 700 confocal scanning microscope (Zeiss, Oberkochen, Germany) and images were processed using Fiji Image J software.

### 4.6. Western Blots

Western blots were carried out as described previously [16,38]. In brief, proteins were transferred to a hybond ECL nitrocellulose membrane using a wet transfer system (Bio-Rad Laboratories, Hercules, CA, USA). Antibodies were diluted in phosphate buffered saline (PBS), 0.05% Tween20, 5% skimmed milk. As for the IFA, the primary α-Ty antibody was a monoclonal antibody (BB2). Secondary antibodies (goat α-mouse, horse radish peroxidase conjugated, Sigma Aldrich, St. Louis, MO, USA; A5278) were visualized using the SuperSignal West Pico PLUS Chemiluminescent Substrate (ThermoFisher Scientific, Waltham, MA, USA; 34580). Images were acquired using the Bio-Rad ChemiDoc MP Imaging System and images were processed using Bio-Rad Image Lab software.

### 4.7. UHPLC-MS/MS Sample Preparation

Parasite cultures were treated for 24 h with 50 nM rapamycin. Five replicates of freshly egressing parasites (2 × 10^8^ parasites, parental line DiCre+/−R, AT6-1-iKD+/−R) were harvested on ice through syringe lysis (27 G needle) and passed through 3 μm exclusion size filters. The metabolism was further quenched through addition of excess (4 volumes) of ice-cold PBS. Parasites were pelleted through centrifugation (2000× *g*, 10 min, 4 °C). Pellets were washed 2 additional times with ice-cold PBS as described previously [21]. Metabolites were extracted in 200 μL of ice-cold acetonitrile (ACN):water (4:1) containing 5 μM ^13^C_6_/^15^N isoleucine (Cambridge Isotope Laboratories, Tewksbury, MA, USA; CNLM-561) as an internal standard. The metabolite extract was dried sequentially in a centrifugal evaporator and resuspended in 60 μL of ultra-pure water. As controls, 6 extraction blanks were prepared (no cells). Samples were immediately frozen and stored at −80 °C until further analyses.

#### U-^13^C Amino Acid Labeling and Sample Preparation

Parasites were harvested as described above, following the down-regulation of AT6-1 through 50 nM rapamycin for 48 h. Five independent replicates were harvested per condition. Purified and washed parasite pellets (2 × 10^8^ cells, DiCre+R, AT6-1-iKD+/−R) were resuspended in 1 mL of Minimal Essential Medium (MEM, Gibco 51200-046; Life Technologies, Carlsbad, CA, USA) supplemented with 5% dialyzed FBS (Pan Biotech, Aidenbach, Germany; P30-2102) and a U-^13^C amino acid mix (Sigma-Aldrich, St. Louis, MO, USA; 426199), with each U-^13^C-amino acid on average at 1 mM. DiCre−R (2 × 10^8^ cells) parasites were harvested as described above but incubated in regular culture medium (no labeling, natural abundance). Parasites were incubated in the respective medium for 1 h at 37 °C in a 15 mL conical tube. The metabolism was rapidly quenched through the addition of 14 mL of ice-cold PBS and placing tubes on ice. Subsequently, cells were harvested, washed with PBS and metabolites were extracted as described above in ACN:water (4:1) containing 40 μM ^13^C_3_/^15^N pantothenate. Samples were not dried but directly frozen and later analyzed as extracts in ACN:water (4:1).

### 4.8. UHPLC-MS/MS Analysis

Metabolites were measured by liquid chromatography mass spectrometry tandem mass spectrometry (UHPLC-MS/MS) using a Q Exactive Plus mass spectrometer coupled to an UltiMate 3000 UHPLC system (ThermoFisher Scientific, Waltham, MA, USA). For reverse-phase separation (RPLC), a Cortecs UPLC T3 column (2.1 × 150 mm, 1.6 µm) and its VanGuard pre-column (Waters, Milford, MA, USA) was used. For HILIC separation, an Acquity UPLC BEH Amide column (2.1 × 150 mm, 1.7 µm) and its VanGuard pre-column (Waters, Milford, MA, USA) was used. RPLC separation was carried out at 250 µL/min with a 0–95%B linear gradient over 20 min (25 min total runtime). Eluent A was 5 mM ammonium formate +0.25% formic acid in ultrapure water and eluent B was ACN. HILIC separation parameters (i.e., gradient and mobile phases) were adapted from Prinsen et al. [43]. The column oven temperature was maintained at 40 °C for both chromatographic modes. Samples were randomized, kept at 6 °C in the autosampler and 5 μL were injected. Blanks were injected between every sample to prevent carry-over.

The mass spectrometer was operated in positive polarity with a heated electrospray ionization (HESI-II) probe. Electrospray voltage was set to 2500 V; the sheath gas, auxiliary gas and sweep gas were set, respectively, to 35, 10 and 1 (arbitrary units, nitrogen). The auxiliary gas heater and transfer capillary temperatures were of 200 °C and 250 °C. For RPLC separation, the looped MS experiments were a full MS1 spectrum experiment (*m/z* 95–850) followed by MS2 data independent acquisition (DIA) using 33 staggered 44 u isolation windows (*m/z* 80–850), each with a normalized collision energy (CE) of 60. All experiments were acquired in profile mode at 17.5 k resolution with an AGC of 1e6 and a fill time of 30 ms (1 µscan). For HILIC separation, the looped MS experiments were a full MS1 spectrum experiment (*m/z* 70–1050) followed by MS2 data independent acquisition (DIA) using 21 staggered 23 u isolation windows (*m/z* 70–300) covering the amino acids mass range, each with a normalized collision energy (CE) of 30. All experiments were acquired in profile mode at 17.5 k resolution with an AGC of 1e6 and a fill time of 50 ms (1 µscan). Xcalibur v.4.2 (ThermoFisher Scientific, Waltham, MA, USA) was used for data acquisition.

### 4.9. MS Data Processing

MS1 data processing of the RPLC dataset was performed using Progenesis QI v.2.4 (Waters-NonlinearDynamics, Milford, MA, USA). Sample alignment and peak picking were performed with an excluded area for *m/z* 675–850 from 12 to 25 min (noise from organic column wash). Sample normalization was performed using all detected features. Singly and doubly charged ions, loss of water as well as Na^+^, NH_4_^+^, K^+^ and ACN adducts were considered for deconvolution. Exported normalized data were further processed in Excel software (Microsoft). MS2-DIA experiments were processed with Skyline-daily v.21.0.9 [44] for identity confirmation using a spectral library built from MS2 fragmentation spectra from authentic standards. For labeling analyses, the abundance of isotopologues for each amino acid was determined using El-Maven [45]. The abundance of each amino acid was determined as the sum of all detected isotopologues, normalized to the internal standard and calculated as relative abundance in comparison to the unlabeled DiCre−R samples, which were incubated in regular DMEM (natural abundance), as described under Parasite Culture and Maintenance. The fraction of ^13^C-labeling was determined, correcting for the occurrence of natural isotopes as described previously [46]. The formula, accurate mass and retention time for each amino acid is given in Table S3.

## Data Availability

Data including raw values are included in the supplementary data. All raw data (MS files) will be made publicly available on a data repository.

## Supplementary Materials

The following are available online at https://www.mdpi.com/article/10.3390/metabo11080476/s1, Table S1: primers, Table S2: untargeted metabolomics data, Table S3: amino acids.
