# Supplementary material for: Untargeted Metabolomics Uncovers the Essential Lysine Transporter in Toxoplasma gondii
Source: Metabolites. 2021 Jul 23;11(8):476. doi: 10.3390/metabo11080476 (PMC8399914; doi:10.3390/metabo11080476)
Supplement: Supplementary file 1 [file metabolites-11-00476-s001.zip › FigureS1.pdf]

Figure S1

(a)

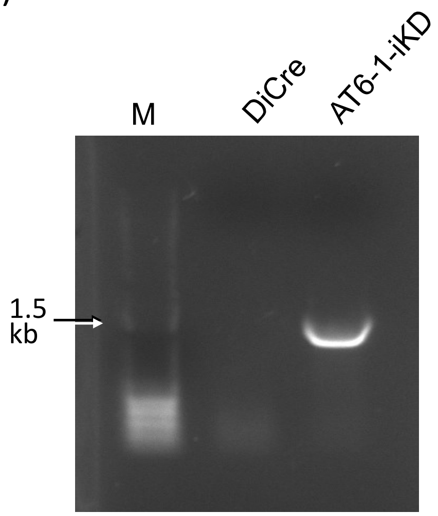

(b)

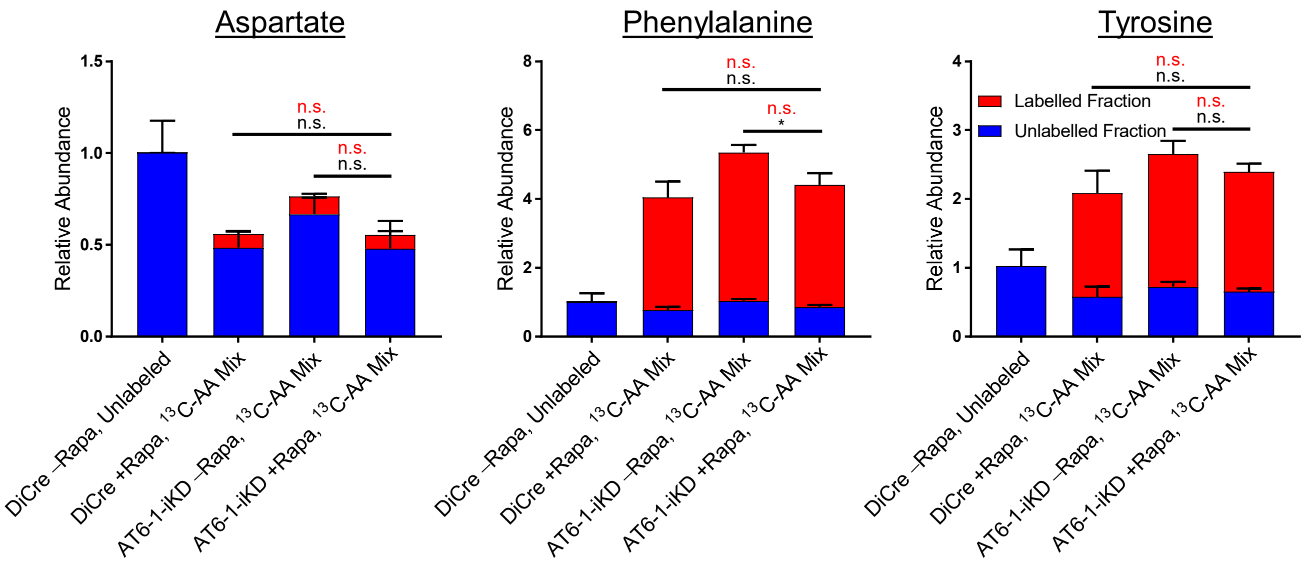

Figure S1. Integration PCR and amino acid labeling. (**a**) A PCR was performed on genomic DNA extracted from DiCre and AT6-1-iKD parasites using primers that anneal in the AT6-1 locus and the HXGPRT resistance cassette, leading to a 1267 bp fragment in the successfully modified strain. (M: base pair/molecular weight marker) (**b**) The relative abundance and fractional ^{13}C-labeling of 3 amino acids (aspartate, negatively charged; phenylalanine, nonpolar and tyrosine, polar) are shown for DiCre−/+ rapamycin (R) parasites and AT6-1-iKD−/+R parasites. DiCre+R parasites were incubated in regular (natural abundance) medium while iΔTgApiAT6-1−/+R parasites and DiCre+R parasites were incubated in medium containing a mix of U-^{13}C-labeled amino acids for 1 hour. The abundance of amino acids was determined as the sum of each amino acid's mass isotopologues normalized to the internal standard and to the relative abundance in DiCre−R parasites (normalized abundance of 1). ^{13}C-labeling was quantified based on the abundance of all mass isotopologues following background correction. Statistically significant differences in abundance (bottom, black) and ^{13}C-labeling (top, red) are indicated (not significant: n.s., **: p-value < 0.001, ***:p-value <0.0001).
